# Extracts from Private Correspondence

**Published:** 1859-03

**Authors:** 


### EXTRACTS FROM PRIVATE CORRESPONDENCE.

*Spirits of Turpentine in Intermittent Fever, and Chloroform in Mania-a-Potu.*—We take the liberty (of which we are sure the writer had no expectation, but for which, we hope he will excuse us,) of publishing some interesting extracts from private letters of Dr. R. A. Fontaine, a promising young physician of Georgetown, Ga. He says: "I must tell you of a happy result obtained in the treatment of an obstinate case of Intermittent, with Spirits of Turpentine. The patient was a gentleman, some twenty five years of age, of feeble habit, and too "ambitious" (vernacular) for his fragile frame. He has been "down" half a dozen times since the commencement of fall and suffers very much; his stomach is so exceedingly irritable that he can retain nothing, more particularly Peruvian bark or any of its derivations, which are his especial aversion. I have given him large injections of the Sulphate of Quinine, but without appreciable effect—finally I recollected the Spirits of Turpentine as recommended by Dr. J. C. Nott, of Mobile, to a lady of my acquaintance, and determined to apply it; accordingly about two hours before the expected attack, I anointed his entire chest, stomach and axillæ with Turpentine, and covered the parts with flannel, thus irritating him to a burning (as he described it) worse than any imagined by Dante. About an hour before the anticipated attack, I gave him a "corn sweat" by boiling a dozen or more ears of corn and placing them while very hot around his body, and still keeping up the Turpentine. He triumphantly escaped, and chills "were free no more" to molest him, for that day at least—he had no rise of fever.

With increasing experience I am more and more convinced that to be a successful practitioner a physician should be "a man of expedients.'"

In another letter he remarks, "One patient afflicted with Mania-a-Potu has given me considerable trouble—he went ten days without food or rest, a prey to the most corroding cares, and surrounded by "bottle imps,"—some of his fantasies would have been truly amusing and ludicrous, but from my knowledge of his true condition and the griefs of an interesting family. I hold it as a self evident axiom in this disease, that unless the patient sleeps, he dies, and consequently gave him thumping doses of opium, solid, powdered and all its derivations, giving not *less* than fifteen or twenty grains in twelve hours, and this, not from bravado, but from what seemed necessity, and that for several days; the cumulative effects of which would have killed a horse. I was surprised that I had not produced narcotism, and thought that the "drowsy God" had forsaken him forever, and that "sleep no more Macbeth" was the order of the day, when it occurred to me to try Chloroform, this I did in the dose of a teaspoonfull, combined with a grain and a half of morphine, and in a half an hour the "balmy restorer" visited him after a ten day's absence. I

sat up with him that night, lest any thing should go wrong, and in the morning, before leaving, was gratified by an almost lucid conversation with him; since then, he has been improving slowly, and his convalescence will be tardy, as his previous excesses had much shattered his constitution."

While we may not agree fully with our young friend in reference to the *extent* of his opium practice, we predict for him a successful career.

